# A Self‐Powered Hydrogel‐Based Wearable Sensor for Sports Motion Monitoring and Activity Recognition

**DOI:** 10.1002/open.70279

**Published:** 2026-08-12

**Authors:** Changqing Miao, Heng Zhao, Xiaojun Wang, Haoxuan Liu

**Affiliations:** ^1^ School of Physical Education Anyang Normal University Anyang China; ^2^ Office of Educational Administration Teaching Research Division Capital University of Physical Education and Sports Beijing China; ^3^ Physical Education Department Henan College of Surveying and Mapping Zhengzhou China

**Keywords:** conductive hydrogel, intelligent evaluation, motion monitoring, self‐powered sensor, triboelectric nanogenerators (TENGs)

## Abstract

Wearable sensors have shown great potential in sports motion monitoring, exercise evaluation, and intelligent training owing to their ability to provide continuous and real‐time biomechanical information. Here, a multinetwork PD hydrogel‐based triboelectric nanogenerator (PD‐TENG) is developed as a self‐powered wearable sensing platform for national fitness motion monitoring and intelligent evaluation. The device adopts a contact–separation configuration with pPTFE and nylon as the triboelectric pair and PD hydrogel as the flexible conductive electrode. Benefiting from the synergistic coupling of polymer chains, ionic species, and dynamic intermolecular interactions, the hydrogel exhibits favorable flexibility, mechanical compliance, and interfacial adaptability. The optimized PD‐TENG delivers an open‐circuit voltage (*V*_OC_) of 876 V, a short‐circuit current (*I*_SC_) of 40 μA, a transferred charge (*Q*_SC_) of 260 nC, and a maximum output power of 598 μW. It also maintains stable electrical performance under varying frequencies, separation distances, temperature, humidity, and external force. As a wearable sensor, it enables joint‐angle monitoring and distinguishes walking, running, and jumping, demonstrating promising potential for intelligent motion recognition and scoring.

## Graphical abstract

A multinetwork poly(vinyl alcohol)/polydopamine hydrogel functions as a compliant ionic electrode in a PTFE–nylon triboelectric nanogenerator. The device reaches 876 V and 40 μA, retains stable output over 36,000 cycles and varied environmental conditions, and responds distinctly to joint bending, walking, running, and jumping, highlighting its potential for self‐powered wearable sports monitoring.

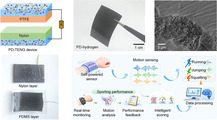

## 1 Introduction

Driven by the rapid progress of next‐generation information and communication technologies, artificial intelligence, and IoT, intelligent sensing is gradually shifting from fragmented and independent signal capture to continuous, interconnected, and high‐accuracy data acquisition [1, 4]. As an important bridge connecting the human body, external surroundings, and digital platforms, wearable electronics have demonstrated broad application prospects in health monitoring, exercise assessment, rehabilitation, human–machine interaction, and intelligent assistive systems [5, 8]. For human motion analysis in particular, sensors are expected not only to faithfully record dynamic biomechanical information, including joint flexion, gait changes, and local deformation, but also to exhibit characteristics such as flexibility, light weight, body conformity, and long‐term wearing comfort, so that they can function reliably on complex skin surfaces during prolonged movement [9, 14]. Compared with traditional rigid electronic components, flexible wearable sensing devices are more advantageous for the real‐time detection and accurate quantification of body motion, thereby offering dependable data for motion identification, condition assessment, and early‐stage health warning [15, 22]. Hence, constructing flexible sensing systems that simultaneously feature high sensitivity, mechanical adaptability, and stable signal output has become a major focus in intelligent wearable electronics research [23, 27].

Although significant progress has been achieved, the majority of existing wearable sensing devices still depend on external batteries, which inevitably add extra volume and mass while constraining device endurance, integration level, and autonomous long‐term operation [28, 31]. In scenarios requiring continuous monitoring of human movement, frequent charging or battery replacement is clearly undesirable for device miniaturization, portability, and large‐scale practical implementation. Under such circumstances, self‐powered sensing technologies have gained growing interest because they can integrate energy harvesting with signal output in a single platform [32, 33]. Among the available approaches, triboelectric nanogenerators (TENGs), based on the synergistic effect of contact electrification and electrostatic induction, are capable of directly transforming low‐frequency mechanical energy from human activity into readable electrical responses [28, 30, 45]. These systems possess several attractive merits, including diverse material choices, versatile structural configurability, low manufacturing cost, and excellent responsiveness to subtle mechanical stimuli [46, 47]. More importantly, TENGs can serve not only as microscale energy harvesters but also as self‐powered sensing units that directly discriminate mechanical variations among different motion patterns, thereby providing an effective route for gait analysis, joint motion monitoring, posture evaluation, and exercise intensity assessment [48, 49]. With further progress in flexible materials, device‐structure engineering, and intelligent signal‐processing methods, TENG‐based self‐powered sensing platforms are expected to play an increasingly prominent role in accurate human motion monitoring and analysis, thus driving wearable electronics toward higher intelligence, sustainability, and integration [50]. Among the flexible materials available for such self‐powered sensing systems, hydrogels have attracted extensive attention due to their natural softness, remarkable stretchability, excellent skin compliance, and adjustable ionic/electrical conductivity [51]. However, traditional hydrogel electrodes still commonly exhibit poor mechanical durability and inadequate environmental resistance, which severely limit their long‐term and high‐precision use in wearable motion monitoring applications.

In this work, we present a multinetwork PD hydrogel‐based TENG, termed PD‐TENG, as a self‐powered wearable sensing platform for national fitness motion monitoring and intelligent evaluation. The device is constructed in a contact–separation mode, where polytetrafluoroethylene (PTFE) and nylon are employed as the triboelectric pair and the as‐fabricated PD hydrogel is used as the flexible conductive electrode. Owing to the synergistic integration of the polymer network, ionic species, and dynamic intermolecular interactions, the hydrogel electrode exhibits desirable flexibility, mechanical compliance, and interfacial adaptability, which are highly favorable for stable biomechanical signal acquisition under repeated deformation. The optimized PD‐TENG delivers a high electrical output, including a peak open‐circuit voltage (*V*_OC_) of 876 V, a short‐circuit current (*I*_SC_) of 40 μA, and a transferred charge (*Q*_SC_) of 260 nC, together with a maximum output power of 598 μW, demonstrating efficient mechanical‐to‐electrical energy conversion capability. In addition, the device maintains stable output behavior under different working frequencies and separation distances, shows reliable capacitor‐charging performance, and preserves repeatable electrical responses under varying temperature, humidity, and external force conditions. More importantly, when configured in wearable formats, the PD‐TENG enables angle‐dependent monitoring of finger, wrist, and knee motions, and clearly distinguishes representative exercise states such as walking, running, and jumping. These results establish the PD‐TENG as a promising self‐powered sensing system for continuous motion detection, movement‐state discrimination, and intelligent scoring, offering a practical strategy for the development of national fitness monitoring and intelligent evaluation platforms.

## 2 Methods

### 2.1 Materials

Acrylic acid (AA) was sourced from Jinan Xingdate Biotechnology Co., Ltd, China. Poly(ethylene glycol) diacrylate (PEGDA 700) was supplied by Shanghai Jizhi Biochemical Technology Co., Ltd, China. Dopamine hydrochloride (DA) was procured from Pande (Shanghai) International Trade Co., Ltd, China. Ethylene glycol (EG) was acquired from Shandong Lukong Supply Chain Co., Ltd, China. Zinc chloride (ZnCl_2_) was purchased from Beijing Bailingwei Technology Co., Ltd, China. Ammonium persulfate (APS) was provided by Jinan Jingyu Chemical Co., Ltd, China. Poly(vinyl alcohol) (PVA) was obtained from Guangzhou Xinxi Metallurgical Chemical Co., Ltd, China. PTFE film and nylon film, used as the triboelectric layers for device assembly, were purchased from Guangzhou Furui New Material Technology Co., Ltd, China and Shanghai Bangsu New Materials Co., Ltd, China, respectively. Copper wires serving as external conductive leads were bought from an online mall. Deionized (DI) water was employed in all experiments.

### 2.2 The Preparation Process of PD Hydrogel

The PD hydrogel was prepared through a facile thermal polymerization strategy. First, a deep eutectic solvent (DES) was prepared by mixing ZnCl_2_ and EG at a molar ratio of 1:4. The mixture was magnetically stirred at 60 °C for 30 min until a clear and homogeneous liquid was obtained. In a separate beaker, PVA was dissolved in DI water at 90 °C with continuous stirring to produce a transparent viscous solution. After complete dissolution, the preformed DES was slowly introduced into the PVA solution and mixed thoroughly to ensure uniform distribution. Subsequently, AA was added as the main polymerizable monomer, followed by the incorporation of a PDA nanoparticle dispersion. The resulting precursor was continuously stirred until no visible phase separation was observed. To further strengthen the polymer network, PEGDA 700 was introduced as the crosslinking agent. APS was then added as the thermal initiator immediately before gelation. The final precursor solution was poured into a predesigned mold and thermally polymerized at 80 °C for 60 min to complete the polymerization and formation of the hydrogel network. After gelation, the obtained hydrogel was naturally cooled to room temperature and removed from the mold for subsequent use. A preliminary single‐factor screening was conducted to determine the hydrogel formulation. The tested concentrations were 6–12 wt% PVA, 25–40 wt% AA, 15–30 wt% DES, and 0–0.5 wt% PDA, based on the total precursor mass. PEGDA 700 and APS were varied from 0.25 to 1.0 wt% and 0.5 to 1.5 wt%, respectively, relative to the mass of AA. The formulations were evaluated according to precursor homogeneity, gelation behavior, structural integrity, electrical conductivity, and triboelectric output stability. The selected formulation contained 8 PVA, 35 AA, 25 DES, and 0.3 wt% PDA, with PEGDA 700 and APS added at 0.5 and 1.0 wt% relative to AA, respectively. The as‐prepared PD hydrogel exhibited a uniform brown appearance, good flexibility, and satisfactory conductivity, making it suitable for use as a compliant electrode in the TENG device. Unless otherwise stated, all samples were prepared under ambient laboratory conditions.

### 2.3 The Preparation Process of PD‐TENG Device

The PD‐TENG device was assembled using the as‐prepared PD hydrogel as the flexible conductive electrode and a pair of polymer films as the triboelectric layers. The PTFE and nylon films were cut into identical pieces with dimensions of 40 × 40 mm, providing an effective contact area of 16 cm^2^. The PD hydrogel electrodes had the same lateral dimensions and a thickness of approximately 2 mm. After assembly with the peripheral supporting frame, the overall device dimensions were approximately 50 × 50 mm. Prior to assembly, the surfaces of the triboelectric layers were cleaned with ethanol and dried at room temperature to remove dust and residual contaminants. The PD hydrogel was then shaped into thin sheets of similar size and attached to the backside of each triboelectric film to serve as the compliant electrode. Copper wires were partially embedded into the hydrogel and further fixed to ensure stable electrical output during repeated deformation. Afterward, the PTFE side and nylon side were arranged face‐to‐face in a vertical contact–separation configuration. The two friction layers were carefully aligned to maintain effective and reproducible contact during periodic mechanical excitation. To improve the structural integrity of the device, the edges of the assembled layers were fixed with an insulating supporting frame or flexible adhesive tape, while keeping the central active area exposed for full contact electrification. The total thickness and effective contact area of the device were controlled to achieve reliable operation and mechanical flexibility. For practical use, the fabricated PD‐TENG could be directly mounted onto a substrate or wearable surface depending on the application scenario. During operation, repeated contact and separation between the PTFE and nylon layers generated triboelectric charges, while the PD hydrogel functioned as a deformable electrode to collect and transmit the induced electrical signals. The final device exhibited good flexibility, stable interfacial contact, and satisfactory electrical connection, making it suitable for biomechanical energy harvesting and self‐powered sensing applications.

### 2.4 Characterization and Measurements

The chemical structure and intermolecular interactions of the PD hydrogel were characterized by FTIR. The ionic conductivity of the PD hydrogel was measured at 25 °C using a two‐electrode method. The conductivity was calculated according to *σ *= *L*/(*RA*), where *L* is the distance between the electrodes, *R* is the measured resistance, and *A* is the cross‐sectional area of the hydrogel. Three independently prepared samples were measured, and the results are presented as the mean ± standard deviation. Its surface morphology and internal microstructure were observed by SEM after freeze‐drying and conductive coating. The macroscopic flexibility of the hydrogel was qualitatively evaluated by bending the freestanding sample and observing whether visible fracture occurred. The triboelectric output performance of the PD‐TENG, including *V*_OC_, *I*_SC_, and *Q*_SC_, was measured under periodic contact–separation motion. The output voltage signals were recorded using an oscilloscope, while the current and *Q*_SC_ were collected by an electrometer (Keithley 6514). The effects of working frequency, separation distance, and external load resistance on the electrical output were systematically investigated, and the capacitor‐charging behavior of the device was further evaluated. In addition, the stability of the PD‐TENG was tested under different temperature, humidity, and force conditions, and its durability was assessed by repeated cycling measurements. For wearable sensing tests, the device was attached to the finger, wrist, knee, and foot to monitor joint bending and representative motion states, including walking, running, and jumping. Unless otherwise specified, all measurements were carried out under ambient laboratory conditions. The mechanical excitation was provided by a programmable linear reciprocating platform, and the normal contact force was calibrated using a digital force gauge mounted between the actuator and the device. Unless otherwise stated, the electrical characterization was conducted at a force of 40 N, a frequency of 5 Hz, and a maximum separation distance of 4 mm. For an effective contact area of 16 cm^2^, the applied force corresponded to a nominal pressure of 25 kPa. During the wearable tests, the hydrogel electrodes, copper leads, and electrical connections were insulated from the skin using polymer films and insulating tape. The participant was not included in the electrical circuit, and the reported voltage was the open‐circuit voltage measured across the device terminals rather than a voltage applied to the body. All tests were conducted on intact skin for short periods under laboratory supervision, and the experiments were stopped if sweating, discomfort, or encapsulation displacement occurred.

## 3 Results and Discussion

### 3.1 Schematic Design, Device Architecture, and Wearable Application of PD‐TENG

**FIGURE 1:**
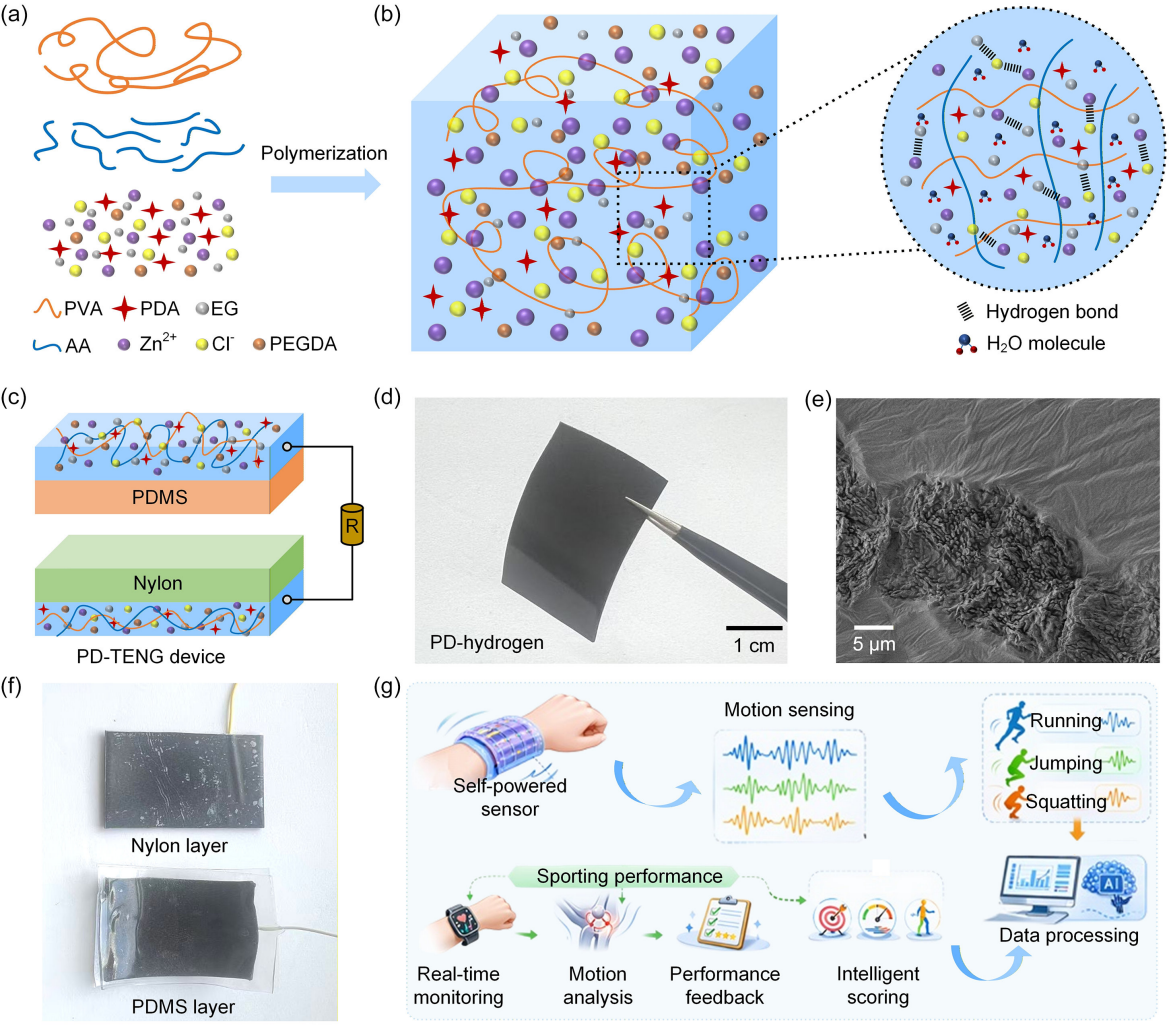
Structural design, device configuration, and wearable sensing application of PD‐TENG. (a) Schematic illustration of the precursor components used for constructing the PD hydrogel. (b) Formation mechanism of the PD hydrogel network after polymerization. (c) Schematic diagram of the PD‐TENG device architecture. (d) Photograph of the as‐prepared PD hydrogel. (e) SEM image of the PD hydrogel surface. (f) Digital photograph of the fabricated PD‐TENG device. (g) Schematic illustration of the wearable application of PD‐TENG.

Figure 1a illustrates the molecular design concept of the PD hydrogel, in which PVA, AA, PDA, EG, Zn^2+^/Cl^−^ ionic species, and PEGDA are introduced as the key building units of the precursor system. The schematic composition not only clarifies the chemical constituents involved in hydrogel construction, but also visually conveys the synergistic integration of polymer chains, ionic components, and functional additives that underpin the final conductive network. Such a formulation lays the foundation for simultaneously achieving mechanical compliance, ionic conductivity, and interfacial adaptability in the hydrogel electrode. Figure 1b presents the polymerization‐induced formation of the three‐dimensional PD hydrogel network. After gelation, the initially discrete components evolve into an interconnected matrix in which long polymer chains are intertwined with abundant ionic species and water molecules. The enlarged view further highlights the presence of dynamic hydrogen‐bonding interactions distributed throughout the network, suggesting that the hydrogel architecture is stabilized not merely by covalent polymerization, but also by reversible supramolecular interactions. This structural feature is particularly important because it endows the hydrogel with both mechanical softness and structural integrity, while preserving continuous ion‐transport pathways for efficient electrical conduction. Figure 1c depicts the structural configuration of the PD‐TENG. The device consists of PTFE and nylon as the triboelectric pair, with the PD hydrogel attached to the backside of each triboelectric layer as a compliant conductive electrode. The two triboelectric layers are arranged in a vertical contact–separation configuration. The schematic clearly demonstrates that the hydrogel electrode is conformally coupled to the backside of each triboelectric layer, enabling effective charge collection during repeated mechanical stimulation. This device architecture is simple yet highly functional, offering a rational route for combining triboelectric generation with a soft, deformable, and conductive hydrogel system. Figure 1d shows the optical image of the as‐prepared PD hydrogel. The freestanding sample can be bent without visible fracture, qualitatively demonstrating its flexibility and film‐forming capability. The uniform appearance of the hydrogel further suggests that the precursor components are well dispersed and that the resulting network is homogeneous at the macroscopic scale. Such mechanical deformability is highly desirable for wearable and biointegrated electronics, where repeated bending and shape adaptation are unavoidable. Figure 1e displays the SEM image of the PD hydrogel microstructure. A rough and interconnected morphology can be observed, implying the formation of a heterogeneous but continuous internal framework. This type of microstructural texture is beneficial for accommodating water molecules and mobile ions, while also contributing to effective stress dissipation under deformation. The microscopic evidence therefore supports the macroscopic flexibility and conductive behavior expected from the hydrogel electrode. Figure 1f provides a photograph of the fabricated PD‐TENG, in which the nylon layer and PTFE layer are separately displayed. The device exhibits an integral, laminated appearance with good structural compactness, demonstrating that the hydrogel electrode can be successfully incorporated into the triboelectric assembly without compromising flexibility. The visible external leads also confirm that the electrical output can be conveniently collected for subsequent performance evaluation. Figure 1g describes the envisioned application scenario of the PD‐TENG as a self‐powered wearable sensor for sports monitoring. Once attached to the human body, the device is capable of converting biomechanical motion into distinct electrical signals, which can be used for real‐time motion sensing and discrimination of activities such as running, jumping, and squatting. More importantly, the generated signals can be further processed for motion analysis, performance feedback, and intelligent scoring, highlighting the strong potential of this PD‐TENG system in data‐driven exercise assessment and next‐generation self‐powered human motion monitoring. The PD hydrogel exhibited an ionic conductivity of 1.21 ± 0.06 S m^−1^ at 25 °C. This conductivity enables effective charge collection and transmission when the hydrogel is employed as the flexible electrode of the PD‐TENG. Because hydrogel conductivity is highly dependent on water content, ionic composition, temperature, sample geometry, and measurement configuration, direct comparison with values obtained under different experimental conditions was not used to rank its performance.

### 3.2 Working Mechanism and Electrical Output Performance of the PD‐TENG

**FIGURE 2:**
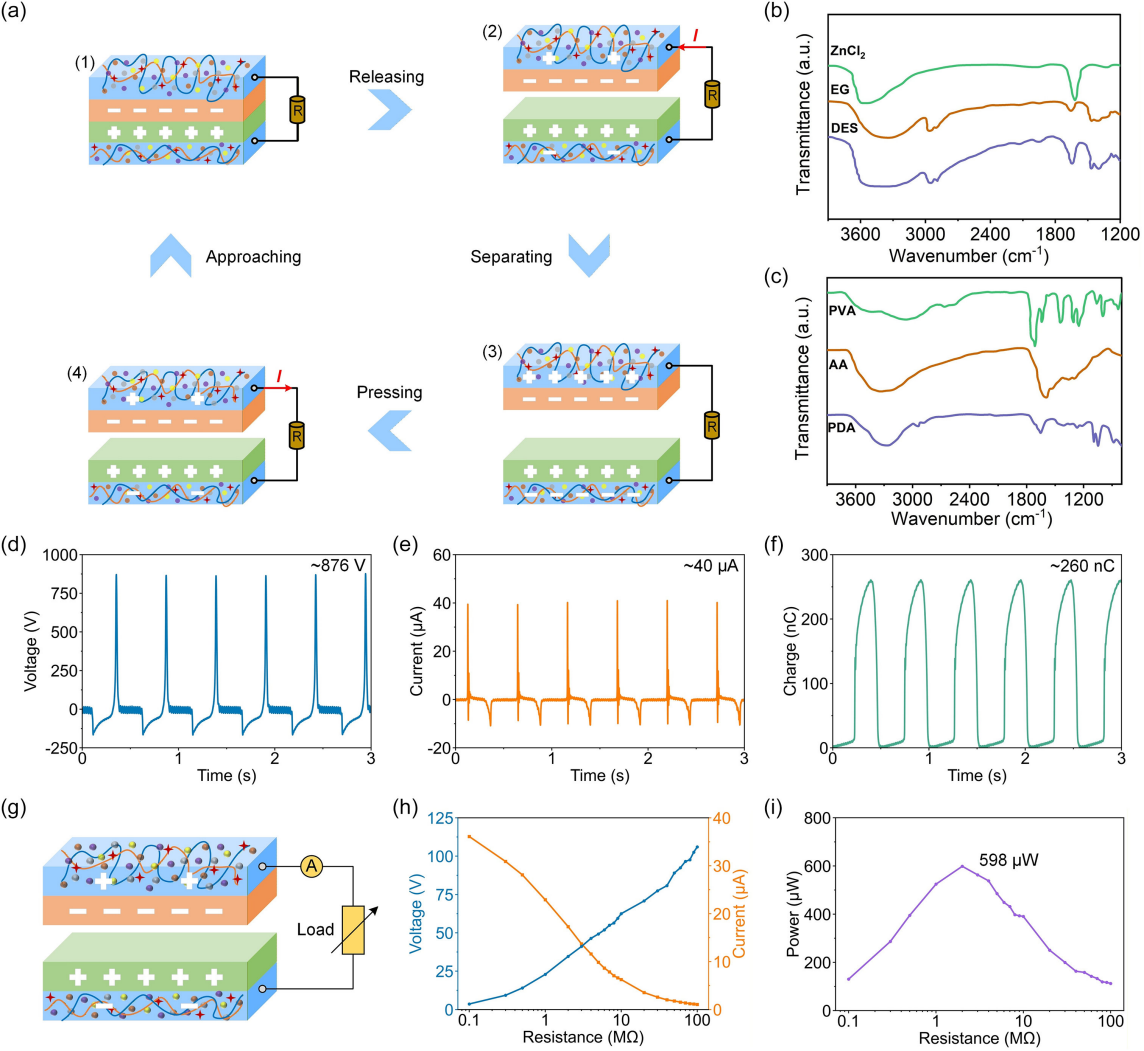
Working mechanism, chemical characterization, and electrical output performance of the PD‐TENG. (a) Schematic illustration of the electricity generation mechanism of the PD‐TENG under a periodic contact–separation process. (b) FTIR spectra of ZnCl_2_, EG, and DES. (c) FTIR spectra of PVA, AA, and PDA. (d) *V*_OC_, (e) *I*_SC_, and (f) *Q*_SC_ of the PD‐TENG during repeated operation. (g) Schematic diagram of the output measurement under different external loads. (h) Dependence of output voltage and current on external resistance. (i) Output power of the PD‐TENG as a function of load resistance.

The electricity‐generation process of the PD‐TENG is illustrated in Figure 2a. When the PTFE and nylon layers are pressed into full contact, opposite triboelectric charges are established on the two friction surfaces because of their different electron affinities. Once the external force is released, the contact interface begins to open, producing a potential difference between the top and bottom electrodes. This potential imbalance drives electrons to move through the outer circuit. As the two layers are separated further, the induced electric field becomes stronger, and the electron transfer process proceeds more completely. During the subsequent recontact stage, the potential difference gradually decreases and then reverses, causing electrons to flow back in the opposite direction. Repeated contact and release therefore generate alternating electrical outputs, which form the basis of the self‐powered sensing behavior of the PD‐TENG. As shown in Figure 2b, EG exhibits a broad O—H stretching band at approximately 3342 cm^−1^. After the formation of the ZnCl_2_/EG DES, this band shifts to approximately 3291 cm^−1^ and becomes broader, corresponding to a red shift of about 51 cm^−1^. This change suggests strengthened coordination and hydrogen‐bonding interactions between the hydroxyl groups of EG and the ZnCl_2_‐derived ionic species. The C—H stretching bands at approximately 2935 and 2875 cm^−1^ remain visible, indicating that the molecular framework of EG is preserved. As shown in Figure 2c, PVA exhibits characteristic O—H and C—H stretching bands at approximately 3305 and 2920 cm^−1^, respectively. AA shows characteristic C=O and C=C bands at approximately 1705 and 1635 cm^−1^, while PDA displays a broad O—H/N—H band near 3350 cm^−1^ and an aromatic C=C vibration at approximately 1510 cm^−1^. These polar functional groups provide potential sites for hydrogen bonding and intermolecular association within the hydrogel network. The spectral changes support the formation of a strongly interacting polar environment that may assist ionic species retention and contribute to network cohesion. However, FTIR alone does not directly demonstrate enhanced ion mobility or mechanical stability. Figure 2c further presents the FTIR spectra of PVA, AA, and PDA. The abundant polar functional groups originating from these components provide favorable conditions for intermolecular association after polymerization, which is important for network formation, ionic conduction, and interfacial adaptability of the hydrogel electrode. It should be emphasized that the FTIR results identify the characteristic functional groups and suggest possible hydrogen‐bonding and coordination interactions, but they do not provide definitive information on elemental bonding states or directly confirm enhanced ion transport. Conventional ex situ surface characterization of water‐rich ionic hydrogels may also be affected by dehydration‐induced ion redistribution and changes in the local coordination environment. Therefore, the proposed molecular interactions are interpreted as being consistent with the observed conductivity and operational stability rather than as being conclusively demonstrated by FTIR alone. The electrical output of the PD‐TENG under cyclic mechanical stimulation is shown in Figure 2d–f. As displayed in Figure 2d, the device generates sharp and highly periodic *V*_OC_ peaks, with a maximum value reaching 876 V, indicating efficient charge separation during repeated contact–separation motion. The corresponding *I*_SC_ signal in Figure 2e exhibits stable pulse‐like characteristics and reaches 40 μA, confirming rapid electron transport through the external circuit. In addition, the *Q*_SC_ response shown in Figure 2f remains regular over continuous cycles and attains 260 nC, demonstrating effective charge accumulation and release during operation. The highly reproducible waveforms in all three cases verify the stable triboelectric conversion capability of the hydrogel‐based device. To evaluate the practical output behavior of the PD‐TENG under different electrical loads, the measurement configuration shown in Figure 2g was employed. As presented in Figure 2h, increasing the external resistance leads to a gradual rise in output voltage but a continuous decline in current, which is consistent with the typical load‐dependent behavior of triboelectric devices. At relatively low resistance, charge transfer is more facile, and current output is favored, whereas high resistance suppresses electron flow and allows greater voltage accumulation across the load. The power–resistance relationship is given in Figure 2i. With increasing load resistance, the output power first rises, reaches a maximum of 598 μW, and then decreases at higher resistance values, revealing an optimal impedance‐matching condition for energy delivery. These results confirm that the PD‐TENG not only exhibits strong self‐powered sensing capability but also possesses attractive potential for powering low‐consumption wearable electronics.

### 3.3 Output Stability and Energy Storage Performance of the PD‐TENG

**FIGURE 3:**
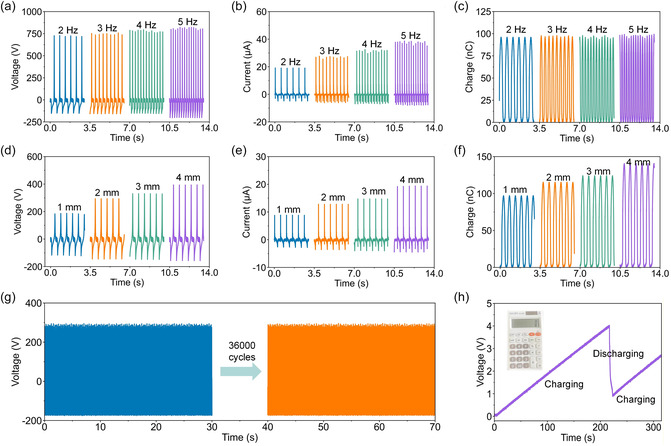
Dynamic electrical response, durability, and energy storage performance of the PD‐TENG. (a) *V*_OC_, (b) *I*_SC_, and (c) *Q*_SC_ of the PD‐TENG under different excitation frequencies. (d) *V*_OC_, (e) *I*_SC_, and (f) *Q*_SC_ of the PD‐TENG at different separation distances. (g) Long‐term durability test of the PD‐TENG over 36,000 operating cycles. (h) Charging and discharging behavior of the PD‐TENG.

Figure 3a shows the voltage output of the PD‐TENG under periodic excitation frequencies ranging from 2 to 5 Hz. As the operating frequency increases, the voltage peaks remain highly regular and well defined, while the peak amplitude exhibits a slight upward trend, rising from the lower level at 2 Hz to a more pronounced output at 5 Hz. This behavior indicates that the device can maintain efficient contact electrification and electrostatic induction even under faster mechanical triggering. More importantly, the waveform morphology remains highly consistent throughout the frequency variation, suggesting that the interfacial contact state and charge generation process are both sufficiently stable to support dynamic biomechanical inputs encountered in realistic motion scenarios. Figure 3b presents the corresponding current response of the PD‐TENG at different excitation frequencies. In contrast to the relatively moderate variation observed in the voltage signal, the current increases more distinctly as the frequency rises from 2 to 5 Hz. This tendency is expected because a higher operating frequency accelerates the charge transfer process within a given time interval, thereby producing sharper and stronger transient current pulses. The excellent periodicity of the current profiles further confirms that the PD hydrogel electrode can effectively collect and transport induced charges under repeated high‐speed actuation, which is particularly desirable for self‐powered sensing systems requiring rapid signal acquisition. Figure 3c displays the transferred charge behavior under the same frequency conditions. Unlike the more frequency‐sensitive current output, the charge signal remains comparatively stable, with only minor fluctuations in peak value as the frequency changes. This result suggests that the total amount of triboelectric charge generated per cycle is primarily governed by the effective interfacial contact area and charge density rather than by the operating speed itself. Such a characteristic is important because it demonstrates that the PD‍‐‍TENG can preserve robust charge generation capability across a relatively broad working‐frequency window, which enhances its adaptability to irregular human motions. Figure 3d shows the voltage output measured under different separation distances, from 1 to 4 mm. As the separation distance increases, the voltage signal rises markedly, and the peak amplitude becomes progressively higher. This phenomenon can be attributed to the enlarged potential difference generated during the contact–separation process when the two triboelectric layers are pulled farther apart. A greater separation distance strengthens the electrostatic field and promotes a larger induced potential across the external circuit, thereby producing a more substantial voltage output. The clear and monotonic increase in peak voltage also implies that the PD‐TENG is highly sensitive to variations in mechanical displacement, which is advantageous for motion amplitude sensing. Figure 3e presents the current response as a function of separation distance. Similar to the trend observed for voltage, the current amplitude also increases as the gap expands from 1 to 4 mm, although the increase is more moderate than that seen in the voltage output. The enhanced current signal indicates that larger displacement facilitates more effective charge transfer during each operating cycle. At the same time, the pulse profiles remain narrow and uniform, revealing that the device can preserve stable electrical transport behavior even when subjected to different mechanical deformation extents. This displacement‐dependent output response further highlights the capability of the PD‐TENG to transduce external mechanical stimuli into discriminable electrical signatures. Figure 3f shows the transferred charge under different separation distances. The charge output exhibits a clear upward trend with increasing gap, reaching the highest value at 4 mm. This result indicates that a larger separation distance enables a more complete electrostatic induction process and improves the effective utilization of triboelectric charges generated at the interface. Because the charge signal is directly related to the amount of transferable surface charge, its systematic increase with separation distance provides strong evidence that the device architecture supports efficient charge harvesting under enhanced mechanical deformation. Figure 3g demonstrates the long‐term durability of the PD‐TENG through a cycling test extending to 36,000 cycles. The stable voltage output over 36,000 contact–separation cycles demonstrates the operational durability of the assembled PD‍‐‍TENG. It should be noted that this test evaluates device‐level electrical stability under repeated contact–separation and does not represent the intrinsic tensile fatigue behavior of the isolated hydrogel. Figure 3h presents the charging–discharging behavior of the PD‐TENG when connected to an energy‐storage unit. The voltage gradually increases with time during the charging stage, indicating that the electrical energy harvested from periodic mechanical excitation can be effectively accumulated. After reaching a higher voltage level, the stored energy is released during the discharging process, followed by another charging stage, which confirms the reversible and practical energy‐storage capability of the system. The digital image inserted in the panel further strengthens the application relevance of the PD‐TENG by showing its compatibility with a simple electronic component. This result suggests that the device is not limited to signal generation alone, but is also capable of serving as an effective microenergy source for low‐power electronics and integrated wearable systems.

### 3.4 Environmental Stability and Force‐Dependent Output Performance of the PD‐TENG

**FIGURE 4:**
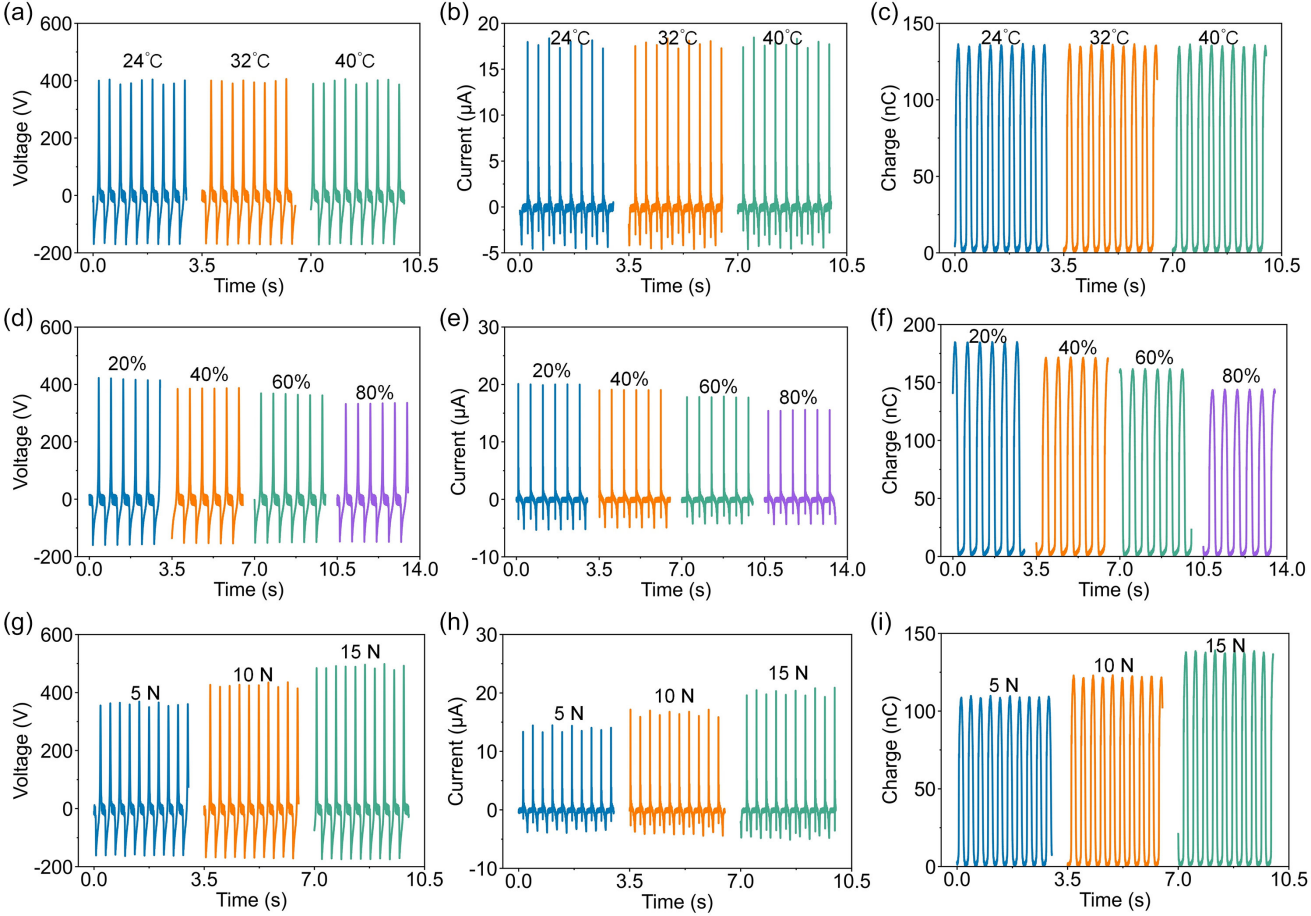
Environmental adaptability and force‐dependent electrical response of the PD‐TENG. (a) *V*_OC_, (b) *I*_SC_, and (c) *Q*_SC_ of the PD‐TENG under different temperatures. (d) *V*_OC_, (e) *I*_SC_, and (f) *Q*_SC_ of the PD‐TENG under different RH conditions. (g) *V*_OC_, (h) *I*_SC_, and (i) *Q*_SC_ of the PD‐TENG under different applied forces.

Figure 4a displays the *V*_OC_ outputs of the PD‐TENG measured at different temperatures. The device delivers highly regular and well‐defined voltage waveforms throughout the tested temperature range, while the peak intensity remains nearly unchanged. This result indicates that moderate thermal variation exerts only a limited influence on the triboelectric charge generation process. The preservation of stable voltage output further suggests that the PD hydrogel electrode maintains reliable conductivity and interfacial compatibility under varying thermal conditions, which is highly advantageous for wearable operation. Figure 4b presents the corresponding *I*_SC_ responses under different temperatures. Similar to the voltage behavior, the current output shows excellent periodicity and only slight variation as the temperature increases. The stable pulse profiles demonstrate that the charge transport process through the hydrogel‐based electrode is not significantly disturbed by thermal fluctuation. Such behavior highlights the desirable thermal tolerance of the PD‐TENG for practical applications involving changing ambient or body‐surface temperatures. Figure 4c shows the transferred charge signals collected at different temperatures. The charge output remains highly stable across the investigated thermal window, with only minimal fluctuation in waveform amplitude. Since the transferred charge directly reflects the effective amount of triboelectric charge generated and exchanged during each working cycle, this result confirms that the core energy‐conversion ability of the device is well preserved under moderate temperature variation. Figure 4d depicts the *V*_OC_ outputs under different RH conditions. A clear downward trend can be observed with increasing RH, indicating that the voltage output gradually weakens in a more humid environment. This behavior can be attributed to the adsorption of moisture on the triboelectric surfaces, which tends to screen surface charges and accelerate charge dissipation. Even so, the voltage waveforms remain periodic and distinguishable, revealing that the PD‐TENG still preserves an effective electrical response under high humidity. Figure 4e presents the *I*_SC_ outputs under different RH conditions. The current signal also decreases progressively as RH rises, which is consistent with the variation observed in the voltage output. The gradual attenuation of current implies that the charge induction and transfer processes are partially suppressed by environmental moisture. Nevertheless, the output remains highly regular over the full humidity range, demonstrating that the device does not suffer from abrupt instability even in relatively humid surroundings. Figure 4f displays the *Q*_SC_ under different RH levels. The charge output shows a continuous decline with increasing humidity, further confirming that moisture weakens the retention and transfer of interfacial charges. Despite this reduction, the charge waveforms remain clear and repeatable, suggesting that the PD‐TENG retains functional operation across a broad RH range. This environmental adaptability is particularly meaningful for practical wearable applications, where humidity changes are often unavoidable. During the frequency‐dependent measurements, the force and separation distance were fixed at 40 N and 4 mm, respectively. During the separation‐distance measurements, the force and frequency were maintained at 40 N and 5 Hz, respectively. For the force‐dependent tests, the applied forces were varied from 10 to 50 N, corresponding to nominal pressures of 6.25–31.25 kPa. Figure 4g shows the *V*_OC_ responses of the PD‐TENG under different external forces. As the applied force increases, the voltage output exhibits an obvious enhancement. This trend indicates that stronger mechanical loading improves the interfacial contact between the triboelectric layers, thereby promoting more efficient charge generation. The well‐separated and highly reproducible voltage signals also suggest that the device is capable of sensitively distinguishing different loading conditions. Figure 4h presents the *I*_SC_ outputs under different applied forces. The current peaks increase steadily with increasing force, revealing that stronger compression facilitates more effective charge transfer through the external circuit. At the same time, the current waveforms remain stable and periodic under all force levels, indicating that the PD hydrogel electrode can accommodate repeated deformation while preserving efficient electrical conduction. Figure 4i displays the *Q*_SC_ under different forces. A distinct increasing trend is observed as the applied load rises, indicating that enhanced contact pressure leads to more effective charge accumulation and transfer during each operating cycle. The clear separation between the charge signals obtained under different forces further demonstrates the excellent force‐dependent response of the PD‐TENG, highlighting its strong potential for self‐powered pressure sensing and motion‐state recognition. The force‐dependent measurements were conducted within the range of 5–15 N to represent moderate mechanical loading associated with joint and limb movements. The present results demonstrate a distinguishable output trend within this range but do not establish the minimum detection limit or sensitivity of the device below 5 N. Evaluation of the low‐force regime requires a mechanical platform with higher force‐control resolution and remains a subject for further investigation.

### 3.5 Self‐Powered Wearable Motion Monitoring and Activity‐State Differentiation

**FIGURE 5:**
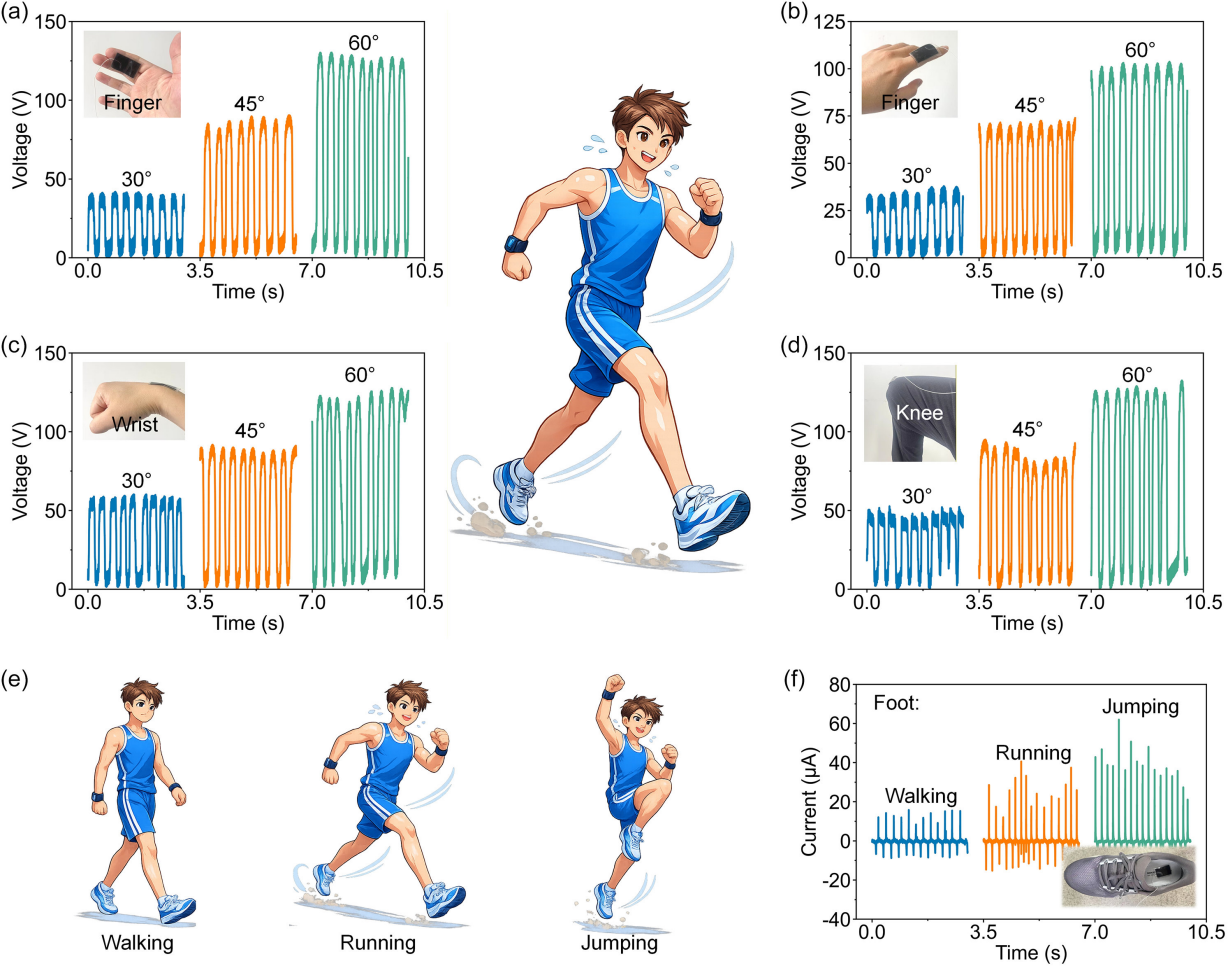
Self‐powered wearable motion recognition and intelligent scoring for sports teaching quality evaluation based on PD‐TENG. (a) Voltage outputs of the PD‐TENG attached to the finger under different bending angles. (b) Voltage responses of the PD‐TENG under another finger‐bending mode at different flexion angles. (c) Voltage outputs of the PD‐TENG mounted on the wrist under different bending angles. (d) Voltage responses of the PD‐TENG attached to the knee under different bending angles. (e) Schematic illustrations of representative sports motions, including walking, running, and jumping. (f) Current outputs of the PD‐TENG integrated at the foot for distinguishing different motion states.

Figure 5a presents the voltage response of the PD‐TENG attached to the finger under different bending angles. As the flexion angle increases from a small deflection to a larger deformation, the output signal exhibits a clear and progressive enhancement, while the waveform remains highly periodic and well separated among different motion states. This angle‐dependent electrical response demonstrates that the PD‐TENG can sensitively transduce subtle finger movements into distinguishable self‐powered signals without relying on an external power source. Such a feature is particularly meaningful for sports teaching scenarios because fine hand and finger actions are often associated with skill standardization, gesture correction, and movement‐detail evaluation in training practice. Figure 5b further shows the voltage outputs generated from another finger‐mounted configuration under different bending angles. Similar to the behavior observed in Figure 5a, the signal intensity increases continuously with increasing flexion, indicating that the device possesses excellent reproducibility and adaptability across different wearing positions. More importantly, the stable response under repeated motion suggests that the PD‐TENG is not limited to single‐point demonstration but can serve as a practical wearable sensing unit for monitoring dynamic joint behavior during sports actions. In the context of intelligent physical education, this capability provides an effective pathway for capturing action amplitude and execution consistency, thereby supporting data‐driven movement recognition and quantitative teaching assessment. Figure 5c displays the voltage response of the PD‐TENG mounted on the wrist under different bending angles. With increasing wrist deflection, the output waveform becomes more pronounced and maintains excellent regularity throughout the testing process. Since wrist posture directly influences the quality of many athletic and instructional movements, including throwing, swinging, gripping, and support actions, the ability of the PD‐TENG to sensitively identify different wrist angles is highly attractive. The result indicates that the device can be employed as a self‐powered wearable node for real‐time monitoring of upper‐limb motion quality, offering objective electrical signatures for subsequent action classification and performance scoring. Figure 5d presents the corresponding voltage outputs obtained from the knee under different bending angles. Compared with finger and wrist motion, knee movement involves larger biomechanical deformation and plays a more direct role in locomotion‐related sports activities. The PD‐TENG still exhibits stable and clearly differentiated signals as the knee angle increases, revealing its strong mechanical compliance and robust sensing capability even under larger joint motion. This behavior is highly relevant to sports teaching quality evaluation because knee flexion is one of the most critical biomechanical indicators in movement standardization for walking, running, jumping, squatting, and lower‐limb coordination training. The distinct electrical response generated at different bending states indicates that the PD‐TENG can provide reliable sensory evidence for identifying movement depth, posture control, and action quality. Figure 5e illustrates three representative whole‐body motion patterns, namely walking, running, and jumping. These schematic or visualized action categories establish a direct link between localized biomechanical sensing and broader sports‐performance evaluation. In practical application, such different exercise modes involve distinct frequencies, amplitudes, impact intensities, and lower‐limb coordination features, which can be effectively reflected in the output behavior of a wearable TENG sensor. By embedding the PD‐TENG into human‐joint or foot‐related positions, the device is capable of converting these complex body motions into structured electrical data, thereby creating the basis for motion recognition, action discrimination, and intelligent scoring in physical education and training environments.

Figure 5f shows the current outputs of the PD‐TENG integrated at the foot during walking, running, and jumping. The three motion modes generate well‐distinguished signal patterns, with progressively enhanced output characteristics as the movement becomes more intense. Walking produces relatively gentle and regular pulses, reflecting a lower‐impact periodic gait. Running leads to stronger and denser current signals, indicating increased step frequency and more vigorous biomechanical input. Jumping generates the most pronounced response, which can be attributed to the higher impact force and rapid release during takeoff and landing. This clear hierarchy in self‐powered electrical output demonstrates that the PD‐TENG is highly effective in differentiating exercise states with different mechanical characteristics. More importantly, the result shown in Figure 5f highlights the application value of the PD‐TENG beyond simple sensing demonstration. Because the device can produce self‐sustained, motion‐dependent electrical signatures directly from human activity, it provides an energy‐autonomous sensing strategy for intelligent sports education systems. When combined with signal‐processing algorithms, these output differences can be further be converted into objective indicators for action recognition, movement standardization, exercise‐intensity evaluation, and training‐effect scoring. Therefore, the PD‐TENG offers a promising route toward self‐powered wearable platforms capable of supporting real‐time sports teaching quality assessment, personalized performance feedback, and intelligent evaluation of physical training behaviors. The distinct electrical responses generated during walking, running, and jumping demonstrate the capability of the PD‐TENG to differentiate representative motion states based on their amplitude and temporal characteristics. These self‐powered signals may serve as input features for future quantitative assessment systems. However, the establishment of a validated scoring model requires a reference‐motion database, feature‐extraction procedures, scoring criteria, and testing across multiple participants, which are beyond the scope of the present proof‐of‐concept study. Integration with a portable microcontroller‐based wireless platform would require a high‐input‐impedance signal‐conditioning circuit, input protection, voltage attenuation, and level conversion before analog‐to‐digital acquisition. Such system‐level integration was not implemented in the present study and remains a direction for future development.

## 4 Conclusion

In summary, a multinetwork PD hydrogel‐based PD‐TENG was successfully developed as a self‐powered wearable sensing platform for national fitness motion monitoring and intelligent evaluation. By integrating PTFE and nylon as the triboelectric pair with the as‐prepared PD hydrogel as the flexible conductive electrode, the device achieved efficient conversion of biomechanical energy into electrical signals. Benefiting from the synergistic effects of the polymer framework, ionic species, and dynamic intermolecular interactions, the PD hydrogel exhibited favorable flexibility, mechanical adaptability, and interfacial compliance, which enabled stable electrical output under repeated deformation. The optimized PD‐TENG delivered high output performance, including a peak *V*_OC_ of 876 V, an *I*_SC_ of 40 μA, a *Q*_SC_ of 260 nC, and a maximum output power of 598 μW. In addition, the device maintained reliable output behavior under different frequencies, separation distances, temperatures, humidity conditions, and applied forces, while also demonstrating long‐term cycling stability and effective capacitor‐charging capability. More importantly, when configured as a wearable sensor, the PD‐TENG realized angle‐dependent monitoring of finger, wrist, and knee motions and clearly discriminated representative exercise states such as walking, running, and jumping. These results indicate that the PD‐TENG provides a promising route toward self‐powered motion sensing, human activity recognition, and intelligent scoring and offers practical potential for the construction of advanced wearable platforms for sports monitoring and national fitness evaluation.

## Conflicts of Interest

The authors declare no conflicts of interest.

## Data Availability

The data that support the findings of this study are available from the corresponding author upon reasonable request.
